# Ethical pharmaceutical promotion and communications worldwide: codes and regulations

**DOI:** 10.1186/1747-5341-9-7

**Published:** 2014-03-29

**Authors:** Jeffrey Francer, Jose Zamarriego Izquierdo, Tamara Music, Kirti Narsai, Chrisoula Nikidis, Heather Simmonds, Paul Woods

**Affiliations:** 1 Pharmaceutical Research and Manufacturers of America (PhRMA), Washington DC, USA; 2 The National Association of the Pharmaceutical Industry in Spain (Farmaindustria) Code Surveillance Unit, Madrid, Spain; 3 International Federation of Pharmaceutical Manufacturers and Associations (IFPMA), 15 Chemin Louis-Dunant, PO Box 195, 1211 Geneva, Switzerland; 4 Formally: Pharmaceutical Industry Association of South Africa (PIASA), Johannesburg, South Africa; 5 Canada’s Research-Based Pharmaceutical Companies (Rx&D Canada), Ottawa, Canada; 6 The Association of the British Pharmaceutical Industry (ABPI) Prescription Medicines Code of Practice Authority (PMCPA), London, UK; 7 Paul Woods Compliance Ltd, Macclesfield, UK

**Keywords:** Pharmaceutical industry, Self-regulation, Code compliance, Promotion of medicines

## Abstract

The international pharmaceutical industry has made significant efforts towards ensuring compliant and ethical communication and interaction with physicians and patients. This article presents the current status of the worldwide governance of communication practices by pharmaceutical companies, concentrating on prescription-only medicines. It analyzes legislative, regulatory, and code-based compliance control mechanisms and highlights significant developments, including the 2006 and 2012 revisions of the International Federation of Pharmaceutical Manufacturers and Associations (IFPMA) Code of Practice.

Developments in international controls, largely built upon long-established rules relating to the quality of advertising material, have contributed to clarifying the scope of acceptable company interactions with healthcare professionals. This article aims to provide policy makers, particularly in developing countries, with an overview of the evolution of mechanisms governing the communication practices, such as the distribution of promotional or scientific material and interactions with healthcare stakeholders, relating to prescription-only medicines.

## Introduction

Rational prescribing decisions should be enhanced by the quality of interactions between healthcare providers and the companies that research and develop medicines. The medicines that research-based companies produce and the scientific information they provide to physicians are important components of quality healthcare for patients. With the ever increasing number of treatment options available to patients, healthcare providers need to be kept up to date with the scientific advancements of new medicines. Likewise, providing patients with information relating to medicines may encourage healthcare providers to explore various treatment options in order to best match patient needs. It is important therefore that the information provided by companies is scientifically accurate and fair. Interactions between pharmaceutical companies and healthcare professionals should always be appropriate and support good patient care. With the aim of further supporting these important goals, the global pharmaceutical industry has made significant changes in recent years in the worldwide controls on companies’ interactions with healthcare professionals. This review explores the mechanisms for ensuring the quality of material supplied by international pharmaceutical manufacturers, including product advertising and educational communications.

Information is often categorized as “promotional,” “non-promotional,” or “scientific”; although the distinction between what is “promotional” and “non-promotional” may not always be clear. Promotional information, as some regulators and codes have defined, encompasses advertising and sales material related to particular products, and may be distributed to patients through advertising campaigns or to healthcare professionals by pharmaceutical representatives. Non-promotional material usually focuses on the current state of understanding of certain diseases and is not related to specific products. Scientific information broadly includes the contributions of research and development (R&D) firms to the exchange of scientific information. For example, scientists from pharmaceutical companies may present research data at scientific conferences or publish works in trade- and peer-reviewed journals. It is important to emphasize, however, that the distinctions between and among these categories arguably matters less to patient welfare than the truthfulness and description of the scientific basis of conclusions about medicines. It should also be noted that prescribers receive information about medical products from a wide variety of sources including clinical trial summaries posted by companies on government registries as well as the medical and scientific literature.

**Figure 1:**
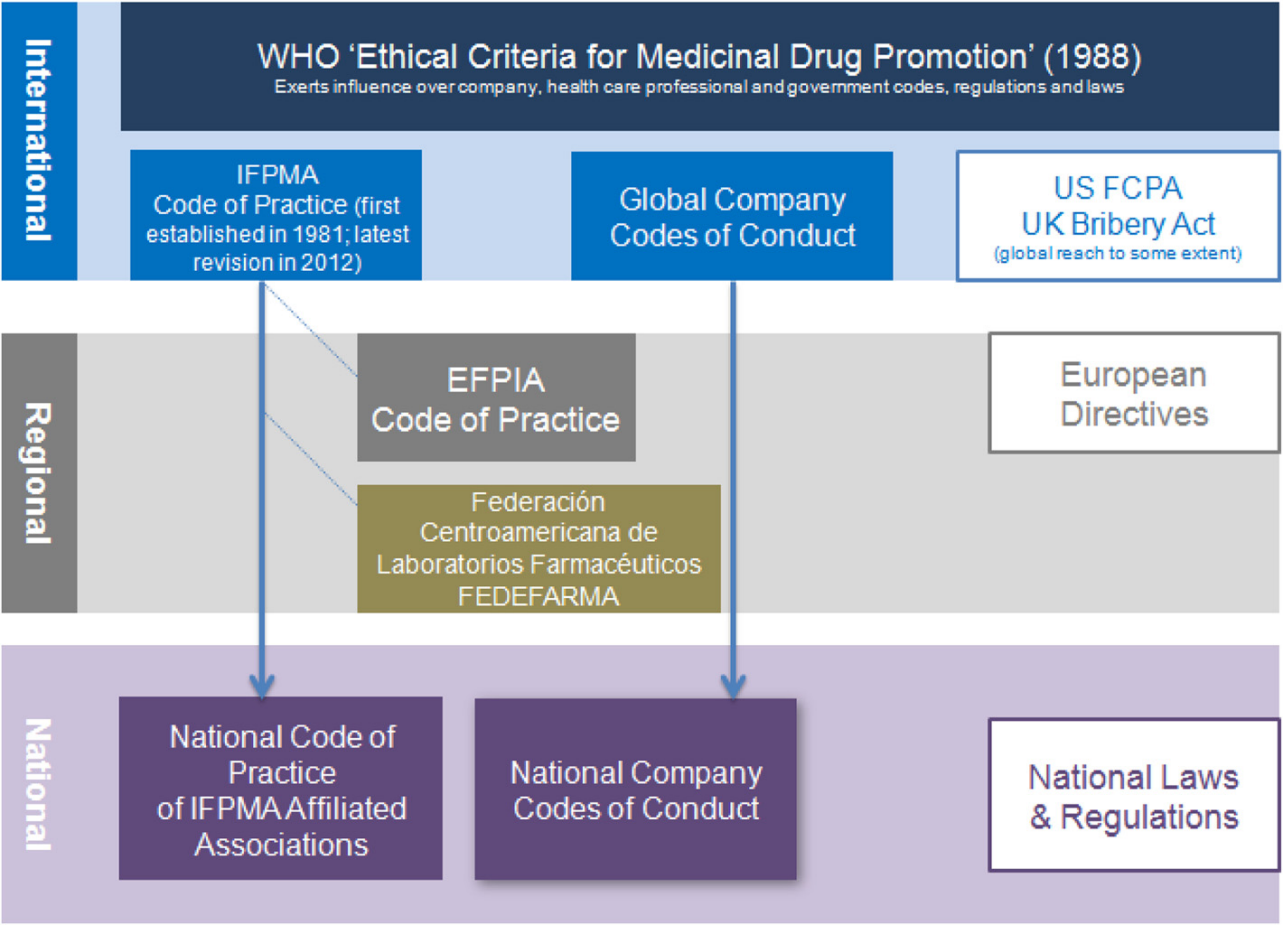
Summary of different code and regulatory mechanisms applying to international pharmaceutical companies.

A range of quality control mechanisms are employed by governments, pharmaceutical companies and industry associations to evaluate the acceptability of companies’ promotional communications and interactions with health professionals (Figure 1). There are systems to take action when concerns are raised and various interest groups also monitor and comment publicly on companies’ promotional activities [1].

Over the past decade, pharmaceutical advertising practices have changed significantly [2]. Laws and regulations have also increased during this period but in many countries the research-based pharmaceutical industry has implemented various mechanisms to self-regulate communication and promotional activities which encompass but go beyond statutory legal requirements. For example, many pharmaceutical companies have substantially expanded compliance functions to ensure that interactions and communications with healthcare professionals and patients are appropriate. Many compliance departments issue internal standards and operating procedures that guide employees’ communications activity and employees are trained on these internal requirements on a regular basis. The goal is to supply healthcare providers with the threshold amount of information needed for informed treatment decisions.

Companies’ standards and operating procedures relating to communications often go beyond the requirements imposed by laws and regulations. However, no compliance documentation can cover all possible situations. Corporate culture is a key aspect of successful self-regulation. When employees understand that communication activities are for the benefit and welfare of patients, the rules governing these activities are put in context. Compliance departments can thus play a critical role in educating and shaping a company’s values and culture.

Analyzing communication activities requires proper identification of the parties involved. Commentary often attributes isolated activities to the whole pharmaceutical industry. It is critical to distinguish not only between research-based and generic-based companies, but also between different types of product (i.e. diagnostic kits, medicines, or medical devices). This paper focuses exclusively on the communication activities of research-based pharmaceutical companies in relation to medicines that are prescribed. National and regional differences in medical, business, and cultural attitudes should also be acknowledged.

Current mechanisms governing pharmaceutical communications comprise industry codes of practice, internal company procedures, laws, and regulations and countries have put in place different combinations of governing mechanisms to reflect national circumstances. Depending on the domestic circumstances, countries may adopt policies to address legislative gaps and/or promote self-regulatory mechanisms. The mechanisms should encompass all sectors of the pharmaceutical industry, including domestic manufacturers and generics producers.

This paper will focus on the international code framework and describe the cascade of code provisions into detailed national codes in developed countries. Several nations with emerging markets (China, India, South Africa etc.) are developing control frameworks that reflect and build on experience in Europe and elsewhere. We also highlight good practice models. We hope that this comprehensive review of current codes and regulatory controls will fill a gap in the literature in an area that often generates debate and controversy. We hope that our review will inform the debate as well as providing a sound basis for future legal and code developments worldwide.

## Analysis of the current situation

### Control systems

Four general categories of control systems govern the communications of pharmaceutical companies relating to prescription products: industry codes of practice, internal company standards, laws and regulations (Table 1). The aim of each mechanism is to enable high standards without compromising effective communication from pharmaceutical companies which benefits healthcare providers and their patients. The effectiveness of a single mechanism should not be determined in isolation. For instance, when industry stakeholders create and adhere to robust codes of practice, regulatory frameworks may be less prominent. However, in most instances, striking a balance among these four mechanisms is necessary to ensure good quality, communications.

**Table 1:** Control systems for prescription medicine advertising

	**IFPMA affiliated industry codes of practice**	**Independent local industry codes of practice**	**Professional bodies**’ **codes of practice**	**Regulatory authority activities**	**Legal actions**	**Company standards**
**Description**	National codes incorporate and expand on the IFPMA Code	National codes, developed independently	International or national medical, pharmacy, and nursing bodies have professional behavior codes. Employers may also have codes of conduct	Regulatory authority interprets and applies law and regulations. Can include pre-approval and post-hoc enforcement	Possible breaches of laws and regulation pursued through court action	Companies have codes of conduct and internal compliance and audit organizations to enforce them
**Applicability**	International pharmaceutical member companies wherever they operate. Includes local companies in a few countries	Local companies that belong to the sponsoring trade association or have agreed to comply with the Code	Applied nationally by the professional body	All sectors within the scope of the legislation. Applied nationally	All sectors within the scope of the legislation. Applied nationally	All countries where the company does business
**Comment**	National codes are often detailed and are subject to national laws and regulations. Some countries embrace code based actions more readily than others	Variable in scope and application	Professional codes may include requirements concerning interactions with commercial organizations	Some regulatory authorities are more active than others	Actions may be brought by government bodies or competitor companies. Some countries resort to legal action more readily than others	Internal standards are usually broader in scope than external codes and legislation

Effective control mechanisms should apply to all interacting parties: pharmaceutical companies, healthcare professionals, government officials, patient groups, and others. Applying codes of practice, laws, and regulations to all the parties involved provides additional safeguards to discourage wrongdoing. For example, in Europe, if an inappropriate payment or gift is given or offered by a company or requested or accepted by a healthcare professional, both parties could be penalized [3]. Similar laws exist in the United States [4].

A significant high-level development occurred early in 2014 when the international bodies representing doctors, pharmacists, nurses, patients and the pharmaceutical industry issued a ‘Consensus Framework’ for ethical collaboration [5]. Under the mantra ‘put patients first’ it sets out common elements for interactions between industry and healthcare professionals. Importantly it does not supersede the partner organisations’ tailored, individual codes or guidelines but rather identifies shared principles.

### Laws and regulations

Laws and regulations that apply to communications relating to prescription medicines operate at national and state levels. For instance, European Union member states are required to apply a baseline set of laws, but individual countries may promulgate additional laws relating to pharmaceutical communications [6]. In addition, general business practice laws apply to pharmaceutical companies as they do to all business sectors. In recent years, anti-bribery and anti-corruption laws have significantly impacted pharmaceutical companies’ interactions with healthcare professionals. For example, the US Foreign Corrupt Practices Act [7] or the UK Bribery Act [8] can affect firms’ activities beyond their respective domestic markets, holding companies subject to such laws accountable for wrongdoings abroad.

Most countries have laws and regulations specific to the advertising of medicines. The European Union has a comprehensive set of legal requirements covering the advertising of medicines [3], which are implemented on a national basis. In the US, labeling and advertising of medicines is regulated under statute by the US Food and Drug Administration [9]. Similarly, in countries such as Canada [10] and Australia [11], specific regulations govern pharmaceutical advertising. However, such regulations are found less consistently in emerging markets.

The practical goal of these laws and regulations is to deter improper activities through enforcement measures. Generally, judicial enforcement can expose companies to substantial financial penalties or settlements, acting as a deterrent to similar future activities. However, court action is often lengthy and expensive. Depending on the circumstances, litigants may opt to settle disputes outside of court, thereby forgoing costs associated with litigation. Furthermore, limited data are available to compare the effectiveness of judicial enforcement with self-regulated adjudications.

Laws and regulations may also be enforced by government regulatory bodies. Some countries, including the US [12] and UK [13], have dedicated regulatory enforcement units. These units can investigate possible violations and initiate enforcement proceedings. Some regulatory adjudication can also be sought by third parties. Often, the regulatory enforcement process is faster than judicial action, reducing costs associated with protracted court actions. Even so any enforcement mechanism requires significant investment and this may be one reason why regulatory bodies are often not as robust in developing countries as in developed jurisdictions.

The consequences for violating laws or regulations governing pharmaceutical product communications can vary greatly by country. Some regulatory bodies have adopted proactive measures, such as mandatory pre-launch reviews [14], to facilitate compliant communications of certain communications such as those on newly approved medicines. In the event that regulatory investigation leads to adjudication, settlements may sometimes be reached.

Relying solely on laws and regulations is a reactive approach to guiding proper communication activities. Nevertheless, robust legal and regulatory mechanisms may be especially useful in countries that do not have other control mechanisms (i.e. codes of practice). At the same time, it is important to point out that pharmaceutical companies that are members of the International Federation of Pharmaceutical Manufacturers and Associations (IFPMA) apply national association codes of practice and the IFPMA Code of Practice [15] worldwide, in every market in which they operate, even in the absence of legal or regulatory controls. This means that in some countries international pharmaceutical companies will be subject to codes and legislation with cross-border reach whereas local companies may not be routinely subject to any robust controls on their advertising and related activities.

### Industry codes of practice

A system of integrated international and national codes of practice on advertising prescription medicines applies to many multinational companies (Table 2). National codes of practice, usually operated by local industry trade associations, have been put in place in developed countries and in many developing countries [14]. Various pharmaceutical industry codes have existed for several decades; however, beginning in 2002, we observed a shift in industry attitudes towards communications activity prompting regular revisions to international codes with all dependent national codes being updated and expanded at least as frequently.

**Table 2:** Codes of practice governing pharmaceutical companies

**Country(ies)**	**IFPMA-affiliated responsible organization**	**IFPMA**-**linked national codes** (further information at http://www.ifpma.org) Additional laws, regulations, codes, and guidelines usually apply in each country. In some cases these codes also apply to companies and/or sectors not affiliated to IFPMA
**Global**		
All countries (Applies to international pharmaceutical companies’ activities in countries not listed below)	International Federation of Pharmaceutical Manufacturers and Associations	IFPMA Code of Practice
**Regional**		
Europe	European Federation of Pharmaceutical Industries and Associations	EFPIA Code on the Promotion of Prescription Only Medicines to, and Interactions with, Healthcare Professionals
		EFPIA Code of Practice on Relationships between the Pharmaceutical Industry and Patient Organizations
		EFPIA Code on Disclosure of Transfers of Value from Pharmaceutical Companies to Healthcare Professionals and Healthcare Organisations
Central America	Federación Centroamericana de Laboratorios Farmacéuticos (FEDEFARMA)	Code of Good Practices for the Promotion of Medicines
**National**		
Argentina	Cámara Argentina de Especialidades Medicinales (CAEMe)	Código de Ética CAEMe
Australia	Medicines Australia	Medicines Australia Code of Conduct
Austria	Association of the Austrian	Pharmig code of conduct and code of procedure of the COC committees of experts of the 1st and 2nd instance
	Pharmaceutical Industry (PHARMIG)	
Belarus	AIPM	AIPM Code of Marketing Practice in the Republic of Belarus
	Association of International Pharmaceutical Manufacturers	
Belgium	Pharma.be	Code of Deontology
Brazil	Interfarma	Código de Conduta
Canada	Rx&D	Code of Ethical Practices
Chile	Cámara de la Industria Farmacéutica de Chile (CIF)	Código FIIM de buenas prácticas para la promociónde los medicamentos
China	R&D-based Pharmaceutical Association in China (RDPAC)	Code of Pharmaceutical Marketing Practices
Colombia	Asociación de Laboratorios Farmacéuticos de Investigación y Desarrollo (AFIDRO)	Código de ética
Czech Republic	Asociace inovativního farmaceutického průmyslu (International Association of Pharmaceutical Industries)	Etický Kodex
Denmark	Lägemiddelindustriforeningen (LIF)	Lif’s ethical rules for dialogue and negotiations with decision-makers
Ecuador	Industria Farmacéutica de Investigación e Innovación (IFI)	Código de Ética IFI
Finland	Pharma Industry Finland (PIF)	PIF Code of Ethics
France	Les entreprises du médicament (LEEM)	Dispositions Déontologiques Professionnelles
Germany	Verband Forschender Arzneimittelhersteller e.V. (VFA) (German Association of Research-Based Pharmaceutical Companies)	FSA Code of Conduct on the Collaboration with Healthcare Professionals
		FSA Code of Conduct on the Collaboration with Patient Organizations
Guatemala	Fedefarma: La Federación Centroamericana de Laboratorios Farmacéuticos	Code of Good Practices for the Promotion of Medicines
Hungary	MAGYOSZ Hungarian Pharmaceutical Manufacturers Association	Code of Ethics for Pharmaceutical Communication
India	Organisation of Pharmaceutical Producers of India (OPPI)	OPPI Code of Pharmaceutical Marketing Practices
Hong Kong	Hong Kong Association of the Pharmaceutical Industry (HKAPI)	Code of pharmaceutical marketing practices
Indonesia	International Pharmaceutical Manufacturer Group (IPMG)	IPMG code of Pharmaceutical Marketing Practices
Ireland	Irish Pharmaceutical Healthcare Association (IPHA)	Code of Marketing Practice for the Pharmaceutical Industry
Italy	FARMINDUSTRIA Associazione delle Imprese del Farmaco	Codice deontologico Farmindustria (code of professional conduct)
Japan	Japan Pharmaceutical Manufacturers Association (JPMA)	JPMA Promotion Code for Prescription Drugs
Korea	Korean Research-based Pharmaceutical Industry Association (KRPIA)	KRPIA Fair Competition Code and its working guideline
Malaysia	Pharmaceutical Association of Malaysia (PhAMA)	PhAMA Code of Conduct
Netherlands	NEFARMA vereiniging innovatieve geneesmiddelen Nederland	Code of conduct for pharmaceutical advertising
Norway	Legemiddelindustriforeningen (LMI)	Rules for marketing of medicinal products. Recommended guidelines between the Norwegian Federation of Organizations of Disabled people (FFO) and the Norwegian association of pharmaceutical manufacturers (LMI) for contact and cooperation between patient organizations and the pharmaceutical industry
Peru	ALAFARPE Asociación Nacional de Laboratorios Farmacéuticos	Código IFPMA de prácticas de marketing farmacéutico
Philippines	Pharmaceutical and Healthcare Association of the Philippines (PHAP)	PHAP Code of Pharmaceutical Marketing Practices
Portugal	Associação Portuguesa da Indústria Farmacêutica (APIFARMA)	Código Deontológico para as Práticas Promocionais da Indústria Farmacêutica e para as Interacções com os Profissionais de Saúde
		Código de Conduta para as Relações entre a Indústria Farmacêutica e as Associações de Doentes
Russia	Association of International Pharmaceuticals Manufacturers (AIPM)	Code of Marketing Practices of the Association of International Pharmaceutical Manufacturers (AIPM)
Singapore	Singapore Association of Pharmaceutical Industries (SAPI)	SAPI Code of Marketing Practices
South Africa	Marketing Code Authority	Code of Marketing Practice for the Marketing and promotion of medicines, medical devices and in vitro diagnostics
Spain	FARMAINDUSTRIA: The National Association of the Pharmaceutical Industry in Spain	Spanish Code of Good Practices for the Promotion of Medicines and Interaction with Healthcare Professionals
		Spanish Code of Practice on Relationships between the Pharmaceutical Industry and Patient Organizations
Sweden	Läkemedelsindustriföreningen (LIF )	Ethical rules for the pharmaceutical industry in Sweden
Switzerland	Interpharma	Code of Conduct of the Pharmaceutical Industry in Switzerland (Pharma Code)
	Scienceindustries Switzerland: Business Association Chemistry Pharma Biotech	
Taiwan	International Research-Based Pharmaceutical Manufacturers Association (IRPMA)	IRPMA Code of Practices
Thailand	Pharmaceutical Research and Manufacturers Association (PReMA)	PREMA Code of Sales and Marketing Practices
Turkey	Association of Research-Based Pharmaceutical Companies (AIFD)	Code on Good Promotion Practices for Medicinal Products to, and Interactions with, Healthcare Professionals
United Kingdom	Association of the British Pharmaceutical Industry (ABPI)	Code of Practice for the Pharmaceutical Industry
United States	Pharmaceutical Research and Manufacturers of America (PhRMA)	Code on Interactions with Healthcare Professionals
		Principles on Conduct of Clinical Trials and Communication of Clinical Trial Results
		PhRMA Guiding Principles on Direct to Consumer Advertisements About Prescription Medicines
		PhRMA Principles on Interactions with Patient Organizations

In 2002, the Pharmaceutical Research and Manufacturers of America (PhRMA) substantially updated its national Code. That revision required PhRMA member companies to follow threshold guidelines relating to communication activities between pharmaceutical companies and healthcare professionals in the United States [16]. In order to synchronize national efforts, IFPMA revised its own code in 2006 [17]. The IFPMA Code of Practice, which was updated again in 2012, binds its members to adopt baseline communications standards.

The practical effect of the 2006 revisions to the IFPMA Code was the creation of a multi-tiered self-regulatory scheme. IFPMA members, consisting of companies and national trade associations, are required to adopt the IFPMA Code. Its reach is wide: companies that are not direct IFPMA members may be bound to the same threshold requirements because of their relationship with IFPMA national associations. Importantly, the IFPMA Code outlines minimum requirements. Members are allowed, and encouraged, to promulgate national or company codes that reflect IFPMA Code requirements, national laws and regulations, healthcare system needs, and local corporate cultures. In this manner, individual companies may be subject to various communication requirements through different sets of obligations.

Industry codes of practice are tiered. National codes must be consistent with the international IFPMA Code of Practice. In Europe, national associations that are members of the European Federation of Pharmaceutical Industries and Associations (EFPIA) must ensure that their codes are consistent with EFPIA Codes [18]. National association codes, in turn, require member companies to follow complementary baseline standards and procedures. Because each level sets minimum requirements, national codes are generally more detailed than international codes. Company standards are even more detailed, often reflecting corporate cultures as well as incorporating international and national codes.

Together, the different levels of codes and company procedures, with few exceptions, include complaint-handling mechanisms, whereby information may be submitted to companies or associations to resolve alleged code violations. Since laws and regulations are mirrored in codes of practice, a concern in some countries has been that full transparency of code of practice rulings might lead to “double jeopardy” (i.e. a second case concerning the same matter) which in turn might inhibit the utility of the code adjudication process. The effective operation of codes of practice requires investment of considerable financial and human resources by national associations. This typically involves the employment of full-time staff to administer the code and its implementation. In addition, independent and industry personnel who make up the adjudication panels devote large amounts of time to adjudicate consistently on cases that can be highly complex.

IFPMA member companies and their agents must comply directly with the IFPMA Code and applicable national codes of member associations where such codes exist. However, the global network of IFPMA-affiliated codes of practice that apply to international pharmaceutical companies does not necessarily extend to other participants and organizations in the healthcare system such as physicians, domestic manufacturers, and suppliers of generics and medical devices. Pharmaceutical companies are only covered by the same codes of practice and standards if they are members of the local IFPMA-affiliated national association and thereby agree to abide by the applicable code (as is the case with international pharmaceutical companies). Healthcare professionals may have their own professional codes of practice which focus on high quality patient care. However, they are often not focused on relationships with commercial enterprises and include far less guidance on healthcare professional – industry relationships than is contained in the industry codes. Domestic companies may belong to other associations with local codes and control mechanisms, and there are separate codes covering the advertising of medical devices. Although all sectors will be subject to applicable laws and regulations, these may not be detailed or diligently applied, and may not exist at all in some countries.

#### Scope of activities covered by codes of practice

The codes of practice, laws, and regulations governing the advertising and selling of prescription medicines cover both what companies can claim about their products and the interactions their employees can have with healthcare professionals, medical institutions, patient groups, and other key stakeholders.

The main areas of coverage of international and national codes are listed below. Note that in some countries, certain requirements are covered by legislation rather than codes.

•Fundamental requirements for ethical and professional behavior, putting patients first, compliance with regulations etc.

•Standards for interactions between companies and healthcare professionals

•Sponsorship or support for healthcare professionals’ attendance at meetings and continuing medical education

•Acceptability of venues and locations for meetings

•Fees for service for engagement of healthcare professionals

•Providing promotional aids, samples etc.

•Hospitality limitations

•Standards for promotional information – accuracy, balance, substantiation etc.

•Essential information for advertisements (e.g. prescribing information)

•Prohibition of promotion of unlicensed products and uses

•Electronic communications

•Interactions with patient organizations

•Clinical research and transparency

•Company procedures and responsibilities, including approval and certification arrangements, staff training

•Complaints handling and enforcement arrangements

Additional coverage of these areas is provided in all European and some other national codes:

•Expanded requirements of the above areas

•Prohibition of direct to consumer advertising for prescription-only medicines

•Specific requirements for representatives

•Requirements for public listings of support and/or engagement of healthcare professionals and/or patient groups

•Donations and grants

•Non-interventional studies

•Aspects of market research activities

•Providing educational and support services e.g. therapy review and nurse services

Additional coverage of these areas occurs in one or more individual codes:

•Expanded requirements of the above areas

•Standards for non-promotional medical information to healthcare professionals and/or patients

•Non-promotional information for patients and the public; disease awareness activities

•Interactions with the media, press releases etc.

•Specific requirements for websites, social media etc.

At a national level, the requirements of codes and legislation usually overlap extensively. A promotional claim or an activity that is illegal will also generally breach the local code of practice. In many countries, the code requirements are broader than those in legislation and/or provide more detail on exactly what is and is not acceptable. In other countries, notably the U.S., competition or antitrust law may limit the ability of companies or national associations to dictate joint marketing rules [19]. Accordingly, in such markets, marketing codes may not include formal adjudication procedures. Rather, rules on the advertising of pharmaceuticals are covered extensively in US laws and regulations. In addition to the basic requirements, such as the essential information that must be included in advertisements (prescribing information etc.), rules cover the two main areas of product claims (e.g. concerning effectiveness and tolerability) and interactions with healthcare professionals (e.g. sponsorship and benefits) [20].

#### Product claims

The same basic rules regarding the veracity of promotional claims are enshrined in most national legislation where it is in place, in the IFPMA Code and in national codes. These have been fundamental requirements since the first codes (which preceded legislation) were put in place. The first industry code governing prescribed medicines (i.e. those available on prescription from a qualified health professional) was initiated in the UK in 1958 [21]. Refinements have occurred since then but the requirement persists that promotional claims must be of high quality and consistent with the prescribing information approved by regulatory authorities. This latter aspect has prompted a high proportion of code complaint cases, often from competing companies.

Generally speaking, the IFPMA Code of Practice and national codes require that product claims relating to prescription medicines be accurate, balanced, and up to date. Material must be truthful and not misleading, including misleading by omission and half-truths. For example, claims must strike a balance of the available evidence and cannot provide only “half the picture”. If challenged, a company is obliged to provide data to substantiate its claims. The IFPMA Code includes the concept that material must be “sufficiently complete to enable the recipient to form his or her own opinion of the therapeutic value” of the product. Materials should also “encourage appropriate use” of medicines by presenting information objectively and without exaggeration. These and other specific requirements set a very high standard for claims in advertisements for prescription medicines, including comparative claims.

Direct to consumer advertising (DTCA) is prohibited in most countries that regulate prescription medicines, although the United States and New Zealand are major exceptions. Although the IFPMA Code of Practice sets global standards, it remains silent on DTCA because the code cannot preempt national laws and regulations. At a national level, codes of practice reflect the local legal situation and usually detail the rules and standards for non-promotional communications concerning prescription medicines that companies can make direct to the public or patients. PhRMA in the US has promulgated a set of voluntary standards regarding DTCA, including appropriate risk communication and timing of certain advertising [20].

A universal and important prohibition relates to advertising a medicine, or a new use of an existing medicine, before regulatory marketing authorization is received. Legislation and codes share similar wording on this point; however, distinguishing promotional and non-promotional information remains complicated. Moreover, at least one appeal court in the United States has recognized the right of companies to provide truthful and non-misleading information about unapproved uses of approved drugs; this decision was based on the companies’ First Amendment right of expression [22]. However, different authorities have different perspectives on the dividing line between “promotional” and “non-promotional” information. Even within one country, regulatory bodies may make different decisions, as in a UK case in which the code decision [23] was stricter than that of the government regulatory body [24] with respect to the responsibility of a company for material it sponsored. Furthermore, new communication mechanisms have blurred the line between promotional and non-promotional material because interactions are no longer necessarily face to face. Nonetheless: the exchange of accurate and data-driven scientific information between pharmaceutical companies and medical practitioners and researchers should bring important benefits to patient care.

#### Pharmaceutical industry interactions with healthcare professionals

Interactions and communication between companies that research and manufacturer medicines and the healthcare professionals that prescribe them are important in contributing to the appropriate and effective use of prescription medicines. These relationships are covered by pharmaceutical advertising codes and legislation. Additionally, national bribery and corruption legislation, such as the US Foreign Corrupt Practices Act (FCPA) and the UK Bribery Act, could have potential application to activities in any country for many companies. Ensuring compliance with the IFPMA Code and the affiliated national codes is likely to help ensure compatibility with relevant sections of anti-bribery legislation. Essentially, the code requirements are designed to prohibit inappropriate personal benefit being offered to healthcare professionals and often go beyond the requirements of anti-bribery legislation.

One issue covered by most national codes is whether companies are able to support healthcare professional attendance at medical conferences. While codes in many countries deem it acceptable to sponsor attendance of healthcare professionals at scientific meetings, and cover associated costs such as reasonable travel, accommodation and meals, they also include a number of caveats. In particular, the main purpose of the meeting must be scientific and professional in nature and any refreshments provided must be incidental to that purpose. The venue must be conducive to the scientific or educational purpose, and international travel must be justified by the international nature of the meeting or other logistical or security reasons.

Company sponsorship of healthcare professionals to attend meetings nevertheless remains a topic of debate. Some countries (e.g. the United States and Norway) do not permit direct sponsorship of attendance at scientific meetings (except for medical students in the US), while others (e.g. France) require review of the arrangements by an independent body. Some countries have put other measures in place such as co-payment of expenses. International companies may also impose on themselves policies relating to sponsorship of healthcare professionals that go beyond external rules [2]. This highlights sensitivity over the perception of companies funding attendance at international educational meetings. However, ceasing sponsorship could deny healthcare professionals without access to sufficient funding the opportunity to hear and interact with world leaders in their chosen field, unless alternative funding arrangements are developed or digitally-based specialist educational services are expanded and are feasible in their country. This is particularly important for healthcare professionals from developing counties, where alternative sources of funding may not be available.

Providing low-value branded promotional aids (pens, pads, tongue depressors, antiseptic wipes etc.) has long been a tradition of pharmaceutical, and other, advertising. International rules still permit inexpensive promotional aids, provided they are relevant to the practice of the healthcare professional. However, there is a trend to ban promotional aids altogether and within the past five years the US [16] and UK [25], amongst others, have prohibited branded promotional aids. At least one global company has ceased their distribution worldwide [2]. The rationale for a ban is not that such promotional aids represent a gift that will affect a healthcare professional’s prescribing or purchasing decisions but rather that such items are not conducive to a new relationship built on mutual professional respect. In addition, industry leaders seek to base relationships with healthcare professionals on sharing educational information rather than on provision of items that could be perceived as gifts.

In most parts of the world, it is permissible to provide samples of medicines to healthcare professionals and such samples may improve patient care. However, the situation varies considerably between countries according to local factors. In a number of countries, samples are not permitted at all, while several countries’ industry codes restrict their number, frequency, and the period after launch during which they can be provided [18,26].

#### Code of practice sanctions

Codes of practice operate on a fundamentally different basis to legislation. They do not rely merely on the threat of punitive fines for their effectiveness. Rather, they represent a collective commitment of member companies to behave in a responsible manner. Deviations from the code requirements are dealt with in a variety of ways that must always be consistent with local laws, including anti-trust and anti-competition provisions (Table 3).

**Table 3:** **Summary of code of practice sanctions and provisions**^**a**,**b**^[14]

**Sanction or requirement**	**Comments**
Requirement to cease non-compliant activity	A universal requirement. Often associated with a written undertaking not to repeat the non-compliant or similar activities, claims etc. The company may be required to recover and destroy offending material. Repetition may result in severe penalties.
Publication of the outcome or public reprimand	Undertaken if local legal considerations allow. May consist of detailed reports or more concise summaries. Offending company is usually identified. In some countries, serious offences may be publicised in the medical press.
Monetary penalties	The amount is usually graded according to the number and/or seriousness of the offences, generally from thousands to hundreds of thousands of dollars.
Additional pre-screening requirements	In countries where pre-screening is optional.
Requirement for a formal audit of company procedures	This is particularly useful if a company’s procedures or training may be the cause of a serious or repeated shortcoming.
Suspension or expulsion from membership of the local trade association	Expulsion may mean that the code regulatory system will not apply to the company and external legal and regulatory controls will therefore take effect routinely. Suspension may mean that the company is still required to comply with the national association code.
Issue a corrective communication	This provision is particularly useful if recipients of the material may have been misled. It will be at the expense of the company.

^a^In most countries, regulatory bodies and legal court proceedings provide additional (or exceptionally the only) sanction options.

^b^Not all sanctions are applied in all countries.

In many countries, fines or administrative fees may be levied and there are requirements to cease the activity that caused the breach. However, the effectiveness of sanctions is mainly based on actions that support the voluntary commitment to good behavior, such as public disclosure of the details of the breach, where local laws permit. In circumstances where companies appear not to have demonstrated the necessary commitment to code compliance, or where the breach is particularly serious, they may be suspended or expelled from membership of the local association that administers the code. The self-regulation system therefore relies on a genuine commitment by companies to take the rules seriously. For international companies, this commitment is reflected in their internal control systems governing promotional activities.

### Company controls

Research-based pharmaceutical companies operate internationally and have global company codes of conduct and detailed standards that apply to many of their activities including sales and advertising. These company policies encompass the requirements of applicable national codes, the IFPMA international code, and legal obligations. However, company compliance standards often add another layer of detail and expand further the scope of activities controlled. These internal codes are often available on company websites and many can be accessed through the IFPMA website [27].

Companies also set out approval procedures for their communications. Prior to use, materials and activities are approved by designated individuals who are responsible for checking acceptability against all applicable laws, regulations, and codes. In Europe and several other countries, there must be a final approval of advertising by a designated doctor or pharmacist. In France and Belgium, the “responsible pharmacist” has a legally constituted responsibility for such approvals. In addition to ensuring compliance with regulations and codes, the physicians and pharmacists who certify promotional activities also have a responsibility as healthcare professionals to patient welfare and are of course subject to the codes of conduct of their professional bodies. Compliance with rules and ethics are not always synonymous; an activity can be legal but not ethical, or considered ethical by many but not legal. What is ethical is open to interpretation and the concept of appointing doctors and pharmacists to approve company outputs reflects a responsibility to patient welfare that goes beyond compliance with written standards.

A number of pioneering initiatives in setting new standards and transparency requirements have resulted from individual company actions. Greater transparency on engagements with healthcare professionals has been initiated in the US and Europe by several companies. Adoption by other companies or, indeed by the industry-wide codes of practice often follows. An example of industry-wide adoption has been the recent European Disclosure Code relating to transfers of value from companies to healthcare professionals. [18]

### Complaint procedures

Healthcare professionals, or indeed anyone including members of the public, journalists, activists, and competitor companies who have concerns about pharmaceutical advertising or activities of pharmaceutical companies, may always raise their concerns. There are a number of available options for involving the types of codes described in this article.

#### Contact the company

People with complaints or questions can approach the local company affiliate and/or the international headquarters. Company compliance departments usually welcome concerns being brought to their attention and many run a confidential “contact us” system – often on their website. Contacting the company can be the most rapid means of resolving a concern. Company standards, in general, cover a broader spectrum of activities than external codes and regulations, and may well govern activities not subject to specific external rules. If the company disagrees with the complainant, the complainant may still resort to the other methods detailed below. The complaints are usually kept confidential and do not result in the issue becoming publicly known, which means other companies cannot therefore learn from the case. However, for simple concerns it can be a quick and efficient method of resolution. Intercompany dialogue is also often the first line of approach when one company is concerned about the activities of another, and can lead to rapid resolution of the matter. Although such dialogue is encouraged, care is needed to ensure compliance with competition laws.

#### Contact national code of practice body

Almost all codes of practice have an associated complaints resolution process. This usually involves detailed consideration of the complaint by a panel of people independent of the company concerned, sometimes including practising healthcare professionals and/or regulatory body representatives. In some cases, the process is overseen by lawyers. Code adjudication processes will lead to a judgment on the matter by reference to the relevant code, which will often be broader in scope than the law and regulations. The process for adjudicating complaints varies between countries and the details are often dependent on local legal and regulatory constraints. Where possible under local legislation, full transparency is encouraged by making public details of the complaint and the company concerned. Local codes often cover a wider spectrum of activities than regulations and the IFPMA Code.

#### Contact IFPMA

When national codes cannot be applied; for example, when the company involved is not subject to the local code or no IFPMA-affiliated association exists in the country concerned, and providing the company involved is a direct member of the IFPMA or belongs to an IFPMA-affiliated association in at least one country, the complaint can be processed by the IFPMA. It will be adjudicated under the IFPMA code operating procedure (Article 13), assuming of course that the subject of the complaint is within the scope of the code. The IFPMA Code does not, however, operate as a higher authority that could overturn a decision made under a national code of practice process.

Although the pharmaceutical industry advocates using the available self regulation options there may be occasions when this is not an option for example if a local company is not covered by the various codes (see Developing Economies section below). Also laws and regulations commonly duplicate requirements of the national self-regulatory codes. The involvement of legal and government regulatory processes therefore remains an option if resolution through Code of Practice procedures is not possible or appropriate.

### Developing economies

Most developed nations have well-established legal systems, regulatory agencies and pharmaceutical industry codes that provide effective control of the advertising of prescription medicines. However, these systems may not be available in all developing nations. The situation is further complicated because, unlike in Europe and North America, international pharmaceutical companies may supply only a small proportion of prescription medicines in some countries. This has practical implications because often only the international companies are bound by the worldwide standards set out in the IFPMA Code. As a result, the more detailed provisions in national codes, as well as the complaint resolution mechanisms that may accompany those codes, leave other sectors within the pharmaceutical industry (e.g. many domestic manufacturers) to operate under different standards and possibly less scrutiny. International companies do not, however, view the additional controls imposed by their codes as a disadvantage. In fact, the opposite may be true, with the application of ethical standards of advertising practice seen by many as a positive advantage.

A further complication in our experience relates to culturally different attitudes to raising concerns and complaints. Some societies do not commonly attempt to resolve concerns through formal complaints mechanisms. This could lead to a situation where clear rules and effective control mechanisms exist but they are under-utilized. However, despite the differences in local controls, a broad range of standards is applied uniformly worldwide to multinational pharmaceutical companies. In addition, since these companies are typically active in the US and UK, action could potentially be taken under the US Foreign Corrupt Practices Act and/or the UK Bribery Act if certain inappropriate interactions with healthcare professionals in other countries were suspected.

As China, India, Latin America, and Africa have become the focus of increased business activity for international pharmaceutical companies, it is important to review significant national developments..

In China, research-based international pharmaceutical companies represent a relatively small proportion of the total market, although their presence is growing and several have committed major research and manufacturing investment there. The trade organization (RDPAC) that represents international pharmaceutical companies in China has a code of practice [28] closely based on the IFPMA Code. However, legal controls dominate and the status of the voluntary code remains uncertain. For example, advertisements must be submitted to the Chinese regulatory authorities for approval before being issued [29].

In India, the majority of pharmaceutical companies are national and do not operate in other countries. There are several thousand such companies, which are represented by national trade associations. Although these associations have codes of practice, they are not bound by the standards and procedures set out by the international IFPMA Code. International companies, including some India-based companies that operate internationally, are members of OPPI (Organization of Pharmaceutical Producers of India) and are governed by its advertising code [30], which is closely linked to the IFPMA Code. Recently, there has been a welcome development whereby a single national pharmaceutical promotion code (Unified Code) has been proposed to harmonize standards across pharmaceutical sectors. Modern legislation governing pharmaceutical advertising is lacking although the *Drugs and Magic Remedies* (*Objectionable Advertisements*) *Act*, *1955*[31], is still in force.

In South Africa, the local industry associations, including those representing makers of medical devices, diagnostics, and generics, as well as prescription and over the counter medicines, have produced a joint code [32] in line with legal provisions in the Medicines Act. Implementation began in autumn 2011. An independent enforcement authority, the Marketing Code Authority, has been established under the code, which includes detailed enforcement procedures and the application of extensive and stringent sanctions in cases of code breaches. The Marketing Code Authority is fully operational and includes a certification process for industry professionals. South Africa is a good example of the willingness of all stakeholders to work together.

In Mexico, collaboration between the local and international pharmaceutical industry, medical associations, medical schools, government bodies, and others led to the agreement in 2008 of joint mandatory transparency guidelines. This arose out of the creation in 2005 of a Council of Ethics and Transparency (CETIFARMA) as well as the more restrictive standards for industry business conduct brought about by the 2006 revision of the IFPMA Code. The council is an autonomous and independent body that operates mandatory codes covering ethics and transparency, good promotional practices, and interaction with patient organizations [33]. Compliance with the code is monitored and sanctions can be applied. There is also a voluntary award system, based on an independent evaluation of company compliance, whereby companies are certified for a two-year period, after which they have to be evaluated again.

It can be concluded that several individual emerging nations have made significant advances in the regulation of the advertising of prescription medicines. The examples of unified codes covering different healthcare sectors and of collaborative codes developed with government and healthcare professional organizations represent an approach for emulation across developing and developed nations.

### Recent developments

Industry codes relating to communication practices are often able to be more representative of sound business practices than laws or regulations because codes can be proactively modified to reflect current needs and trends. Since the first codes relating to pharmaceutical communications were adopted in the 1950s, periodic updates have served as a mechanism for addressing the changing national landscape as well as preventing potential incidents in business practices. In recent years, code updates have primarily related to promotional practices and interactions between pharmaceutical companies and other stakeholders. That area reflects the topics of debate and criticism in the lay and medical literature.

### 2006 update to the IFPMA Code

The 2006 update to the IFPMA Code of Pharmaceutical Marketing Practices marked a significant development in communication practices for IFPMA member companies and associations [34]. Overall, that revision simplified the language of the previous code and expanded the rules relating to company interactions with healthcare professionals. In addition, it revised compliance procedures and established a global Code Compliance Network (CCN). Importantly, the 2006 Code reinvigorated the efforts of member companies and associations to raise public awareness of the self-regulatory regime many pharmaceutical companies had adopted.

The revised sections relating to interactions with healthcare professionals provided increased clarity on company-sponsored events, hospitality, and gifts. Company sponsorship of international events, such as congresses, conferences, and symposia, was narrowly limited to educational or scientific purposes. In other words, information relating to pharmaceutical products at such events was limited to providing participants with scientific and educational information. In addition, stricter rules were put in place relating to company-hosted events, requiring them to be held at venues conducive to the scientific or educational purpose of the meeting. It was made clearer that practices such as providing event participants with extravagant meals, trips to exotic locations for meetings, theatre tickets, rounds of golf, or paying for accompanying guests such as spouses, were not acceptable.

Similarly, rules affecting gifts to healthcare professionals were strengthened. Personal gifts such as CDs, DVDs, theatre or sporting tickets, or anything for the personal benefit of a healthcare professional were explicitly banned. Promotional aids of minimal value and relevant to professional practice were still allowed (e.g. branded pens and pads), as were items of medical utility for patient care (e.g. textbooks and anatomical models) and inexpensive and infrequent cultural courtesy gifts such as those given traditionally for significant religious festivals (e.g. moon cakes). Because these new provisions were implemented worldwide, local codes were encouraged to provide details of the costs and types of item that could be offered.

Clarifying the restrictions on hospitality and gifts was significant, but as these rules were applied globally they were not without opposition. In some countries, particularly those where personal relationships form the basis of business relationships, healthcare professionals had become accustomed to companies’ “generosity.” The reasons why such “benefits” were no longer available had to be explained to some healthcare professionals. Those discussions helped to highlight the ethical responsibilities of healthcare professionals and the role they play in ensuring ethical and professional interactions. Such responsibilities are increasingly reflected in healthcare professionals’ codes of conduct [35].

The IFPMA Code Compliance Network (CCN) established by the 2006 revision of the IFPMA Code brings together over 100 compliance experts representing member associations and companies. CCN members meet regularly to discuss the latest developments and issues in the field of ethical promotion of medicines. The goal is to exchange experiences and ensure effective code implementation at local and national levels.

Finally, the 2006 revision included a section that further elaborated on code complaint procedures, outlining procedural and substantive requirements for validation, referral, and adjudication. In addition, the section included members’ obligations in the event a complaint was lodged about them. If they were found be in breach of the Code, given other enumerated circumstances, a short summary describing the complaint and the decision would be made public.

### 2012 update to IFPMA Code

The IFPMA Code was again expanded in 2012. A new title, *The IFPMA Code of Practice* (omitting earlier reference to marketing practices), reflected the extended scope beyond marketing activities. In particular, the 2012 revisions addressed fees for services, clinical research transparency, and interactions with patient organizations. In addition, companies were required to train employees on relevant conduct practices reflected in the Code. Finally, the Code’s comprehensiveness was improved by clarifying certain articles and extending its scope to such issues as the commissioning of advisory boards and support for continuing medical education (CME).

A significant addition was the listing of ‘Guiding Principles’ which identify the underlying principles on which the detailed rules that follow had been based. This was considered useful because no set of code rules can hope to cover all situations and stating underlying principles should help interpretation of individual cases and when the IFPMA minimum standards are incorporated in the more detailed local codes.

Guiding principles of the 2012 IFPMA Code of Practice:

1. The health-care and well-being of patients are the first priority for pharmaceutical companies.

2. Pharmaceutical companies will conform to high standards of quality, safety, and efficacy as determined by regulatory authorities.

3. Pharmaceutical companies’ interactions with stakeholders must at all times be ethical, appropriate, and professional. Nothing should be offered or provided by a company in a manner or on conditions that would have an inappropriate influence.

4. Pharmaceutical companies are responsible for providing accurate, balanced, and scientifically valid data on products.

5. Promotion must be ethical, accurate, balanced, and must not be misleading. Information in promotional materials must support proper assessment of the risks and benefits of the product and its appropriate use.

6. Pharmaceutical companies will respect the privacy and personal information of patients.

7. All clinical trials and scientific research sponsored or supported by companies will be conducted with the intent to develop knowledge that will benefit patients and advance science and medicine. Pharmaceutical companies are committed to the transparency of industry-sponsored clinical trials in patients.

8. Pharmaceutical companies should adhere to applicable industry codes in both the spirit and the letter. To achieve this, pharmaceutical companies will ensure that all relevant personnel are appropriately trained.

Companies’ engagements with healthcare professionals providing consulting services, such as scientific consulting, market research, and advisory board participation, were elaborated in the 2012 revision. The goal was to ensure that contractual relationships are clearly defined and documented.. In part, the Code requires a written contract or agreement and a clear business need for the services provided. Remuneration must reflect fair market value.

Another new section (Article 9) relates to clinical research and transparency. This addition to the Code reflects a long-standing commitment to disclose clinical trial information in line with the joint disclosures issued by international and national industry associations [36]. It emphasizes the industry’s commitment to transparency in clinical research by including it within the mandatory requirements of a code of practice. Similarly, a section on company support for continuing medical education (CME) has been added (Article 10). This section reaffirms that the primary purpose of CME is to enhance medical knowledge and requires any company contributions to content to be fair, balanced, and objective. Similar provisions apply to CME events as to promotional events.

Rules on companies’ interactions with patient organizations, explicit in the 2012 IFPMA Code (Article 11), are for the most part based on European standards [37]. The rules are designed to safeguard the independence of patient organizations and ensure that support by companies is appropriate. The involvement of the company and the nature of that involvement must be clear from the outset. Written documentation must be in place and arrangements for meetings that companies support are subject to similar restrictions to those that apply to healthcare professional meetings.

Certain existing requirements were clarified in the 2012 edition. For instance, entertainment or social activities cannot be provided or funded by companies, whereas previous versions allowed modest entertainment. The operating procedure for handling complaints was expanded, and new standard operating procedures were added. Under the 2012 Code, the outcome of all complaints will be published, although no-breach complaints will not identify the products or company involved. This revision aims to provide stakeholders with additional guidance on acceptable business practices. Similarly, the “Questions and Answers” section has been expanded to provide more detail regarding key provisions of the Code. Nevertheless, because the vast majority of complaints are dealt with through national code procedures, the IFPMA Code complaint system is unlikely to see a big increase in volume.

### Beyond 2012

In recent years many national codes have been updated and expanded [34], including those in Europe (EFPIA) [18], the US (PhRMA) [16], Canada (Rx&D) [38], and Australia (Medicines Australia) [39]. These often have a broader scope than the IFPMA Code. Certain national codes have taken a lead with rules or guidance in new areas such as digital media communications [40,41]. Even so, the 2012 update of the IFPMA Code required each national code to validate its coverage against the expanded global baseline rules. Many already covered the new areas but others needed revision to ensure that national provisions reflect the IFPMA Code.

National or regional code changes do not have a direct effect on requirements outside their jurisdiction (e.g. in many developing countries). However, national associations share their experiences and have driven changes to the IFPMA Code, as have updates by international pharmaceutical companies to their internal codes of conduct, standards, and procedures [42]. These globally operating companies have had to make decisions about what they will commit to worldwide, which may go beyond the already high standards set out in the IFPMA Code and other national codes and regulations. Another important way of evolving requirements is through individual companies taking a lead in particular areas.

The 2014 ‘Consensus Framework for Ethical Collaboration between Patients’ Organisations, Healthcare Professionals and the Pharmaceutical Industry” [5] which the international pharmaceutical industry supported alongside the international representative bodies for doctors, pharmacists, nurses and patients brings together in one document shared principles which can serve as a valuable model for similar initiatives at the national level.

## Conclusions

The attention and resources devoted to regulatory compliance regarding communication about prescription only medicines is probably at an all-time high. However, no set of rules is beyond improvement and changes in health systems, as well as advances in communication technology, will mean that codes, regulations, and laws will continue to evolve if they are to support optimal use of medicines to benefit patients.

Continuing experience with the operation of existing codes will help inform future developments particularly in rapidly developing countries where international pharmaceutical company activities are expanding yet local manufacturers may not be subject to established codes of practice. Future developments in these countries should strive for international harmonization embracing all healthcare sectors but also take into account national differences. and simultaneously encourage broader participation and endorsement of codes across the industry operating in these countries.

The IFPMA Code was extensively revised in 2006 and again in 2012 It is now well-established as an international model for effective local codes. Continued assessment of of national industry codes of practice is is appropriate to ensure that companies continue to meet the needs of patients and prescribers. Additionally individual companies will continue to pioneer additional standards, approaches, and initiatives. Areas that are being addressed at national and company level include a focus on increased transparency of the relationships between companies and both individual healthcare professionals and healthcare organizations. Low cost promotional aids are being increasingly restricted or banned altogether by companies and national or regional codes.

Laws and regulations may change more slowly, but, in countries where there are perceived gaps, we can expect clarification in the form of new regulations and guidance.

The pharmaceutical industry must continue to serve as a trusted partner in healthcare provision. Industry codes of practice can form the foundation for governing companies’ interactions and communications and therefore play an important part in the relationship between companies and other stakeholders in healthcare provision. Laws and regulations will remain important and legal action will be applied when needed. Nevertheless it will be important to avoid a “box-ticking” approach where the only question is “Is it legal to do that?” but rather to also encompass a code-based evaluation that goes beyond legal requirements.

International companies have established global internal standards but they represent only a small share in the healthcare market in many developing countries, and it would be appropriate for unified self-regulatory codes to cover all sectors of the pharmaceutical market. We have already seen such developments in Mexico and South Africa, and such a model has also been proposed in India. A model of cooperation between industry codes and legislation already works well in some countries, particularly in Europe and Australia. Such a model could be equally successful in developing nations.

## Abbreviations

CCN: Code Compliance Network (a committee of IFPMA); CETIFARMA: Council of Ethics and Transparency (Mexico); CME: Continuing Medical Education; DTCA: Direct to Consumer Advertising; EFPIA: European Federation of Pharmaceutical Industries & Associations; FCPA: Foreign Corrupt Practices Act (USA); IFPMA: International Federation of Pharmaceutical Manufacturers and Associations; MCA: Marketing Code Authority (South Africa); OPPI: Organization of Pharmaceutical Producers of India; PhRMA: Pharmaceutical Research and Manufacturers of America (USA); RDPAC: Research & Development-based Pharmaceutical Association Committee (China); R&D: Research and Development; Rx&D: Canada’s Research-Based Pharmaceutical Companies.

## Competing interests

The authors are each employed by companies or organizations related to the pharmaceutical industry and IFPMA. IFPMA member companies research, develop, manufacture and supply biopharmaceutical products and vaccines globally. IFPMA member associations represent the pharmaceutical industry at a national level. As employees in the pharmaceutical industry some authors may have other financial interests in pharmaceutical companies.

## Authors’ contribution

This publication is the product of extensive collaboration between the members of IFPMA’s Code Compliance Network (CCN). All authors contributed content and ideas to the manuscript which was based on an initial draft by PW. All authors read and approved the final manuscript.

## Authors’ information

HS, CN, KN, TM, JZI, JF and PW are current or past members of the Code Compliance Network of the International Federation of Pharmaceutical Manufacturers and Associations (IFPMA CCN) 15 Chemin Louis-Dunant, PO Box 195, 1211 Geneva 20, Switzerland.
